# Supplementary material for: Timely Monitoring of SARS-CoV-2 RNA Fragments in Wastewater Shows the Emergence of JN.1 (BA.2.86.1.1, Clade 23I) in Berlin, Germany
Source: Viruses. 2024 Jan 10;16(1):102. doi: 10.3390/v16010102 (PMC10818819; doi:10.3390/v16010102)

SUPPLEMENTARY

Timely Monitoring of SARS-CoV-2 RNA Fragments in Wastewater Shows the Emergence of JN.1 (BA.2.86.1.1, Clade 23I) in Berlin, Germany

Alexander Bartel[†], José Horacio Grau[†], Julia Bitzegeio, Dirk Werber, Nico Linzner, Vera Schumacher, Sonja Garske, Karsten Liere, Thomas Hackenbeck, Sofia Isabell Rupp, Daniel Sagebiel, Uta Böckelmann, Martin Meixner

Sequencing and analysis of SARS-CoV-2 fragments in wastewater

Each week, one wastewater sample per sampling location was processed for genomic surveillance in order to monitor the circulating SARS-CoV-2 lineages. For the Nucleic acid isolation and the sequencing, two standardized and validated workflows were used. Concentration and purification of nucleic acids were performed using the Wizard Enviro Total Nucleic Acid Kit (Promega GmbH, Gutenbergring 10, 69190 Walldorf). The procedure was carried out as specified by the manufacturer with the following modifications. Approximately 50 ml of each wastewater sample is transferred to a Falcon tube and centrifuged for 20 minutes at 4°C and 4500 rpm. Then 40 ml of the supernatant are transferred using a syringe filter (0,45 µm, PES membrane) into a new Falcon Tube. Amplification was done using V3 and V4.1 ARTIC primer sets, targeted library preparation and sequencing of the complete SARS-CoV-2 genome with short reads employing the illumina COVIDSeq Test (illumina Inc., Madison WI, U.S.A.). Sequencing was performed on a NextSeq 550 machine using TG Next Seq 500/550 High Output Kits v.2.5 (75 Cycles) in a 2x 35 b run modus. BAM files were converted to FASTQ by using SAMtools bam2fastq. Distinctive mutations associated with COVID-19 clades were carefully chosen as independent markers for precise detection. The NextStrain and GISAID databases provided a reference for SARS-CoV-2 variants in Europe. Reads were mapped on to the SARS-CoV-2, via a bowtie2-samtools pipeline. Single nucleotide polymorphism (SNP) were extracted from bam files using deepSNV R package (version 1.46.0). Relative SARS-CoV-2 lineage abundance estimation using Freyja (v1.3.12; https://github.com/andersen-lab/Freyja; curated lineage file and UShER global phylogenetic tree downloaded on 2023-12-09).

Region	October 2022	October 2023
Worldwide	307.917	83.287
Germany	24.115	1.401
Berlin	944	220

Table S1. Comparison of sequencing efforts between October 2022 and October 2023. GISAID submissions of the SARS-Cov-2 entries collected from humans with complete date collection. Accessed 11th December 2023.

TableS2. Timespan between Date of Sampling to reporting of SARS-CoV-2 sequence to the national integrated genomic surveillance database KI in days

RKI sequence data for Germany since 01.08.2023

Time differences in days

10%	20%	30%	40%	50%	60%	70%	80%	90%
13.0	13.4	14.0	14.0	14.5	17.0	18.0	19.6	21.9

Source: https://github.com/robert-koch-institut/SARS-CoV-2-Sequenzdaten_aus_Deutschland

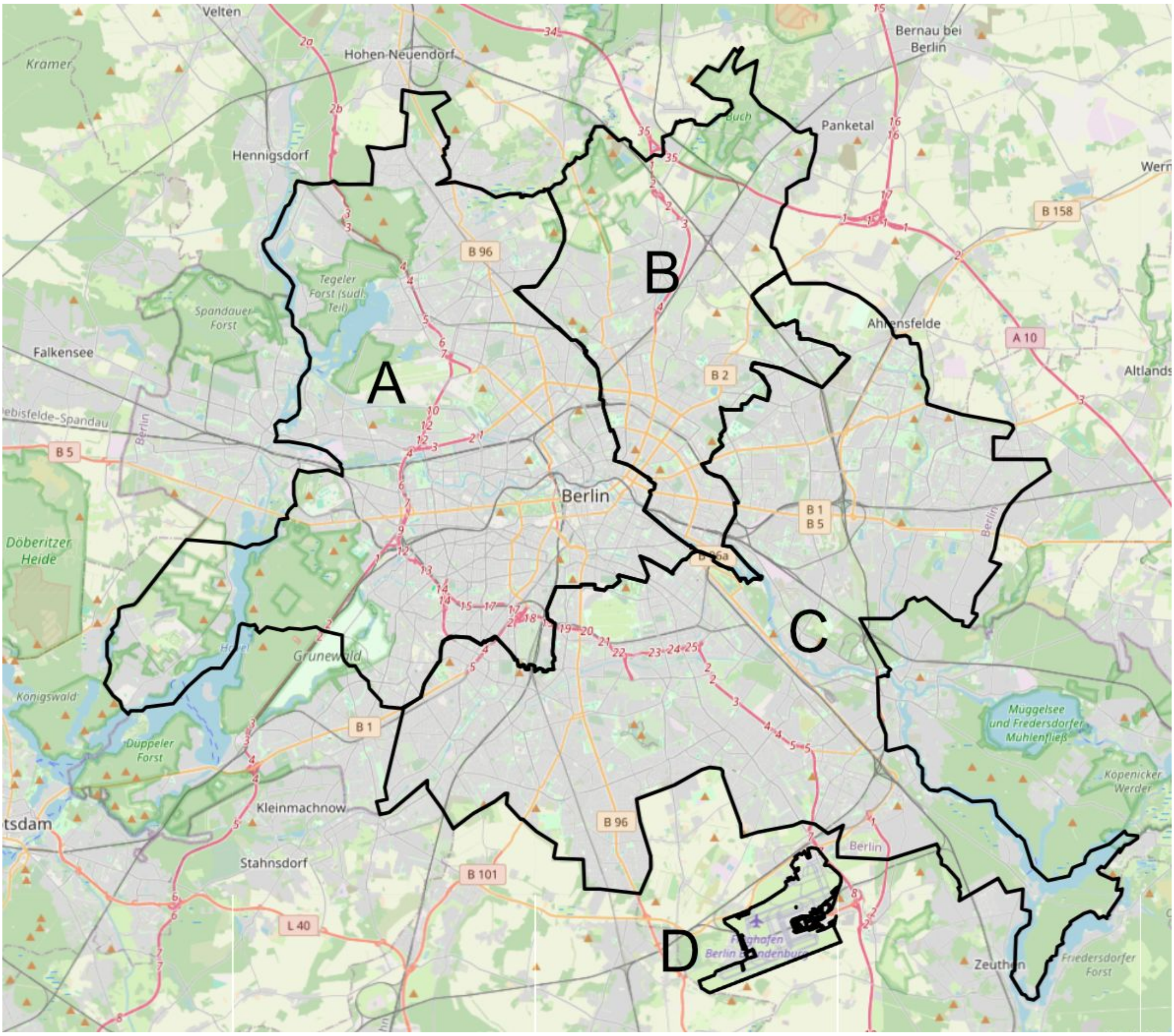

Figure S1. Wastewater monitoring in Berlin Germany is performed in 3 wastewater treatment plants (WWTP) and at the BER airport. A) WWTP Ruhleben, B) WWTP Schönerlinde, C) WWTP Waßmanndorf. We sequenced 4 regions of Berlin which included 97 samples, totalling 1.62 billion reads with on average 63% of the genome covered over 100x in each sample (coverage score). A) WWTP Ruhleben, 24 samples, 254 million, coverage score 59. B) WWTP Schönerlinde, 26 samples, 515 million reads, coverage score 67. C) WWTP Waßmannsdorf, 23 samples, 277 million reads, coverage score 43. D) BER Airport, 24 samples, 573 million reads, mean coverage score 80.

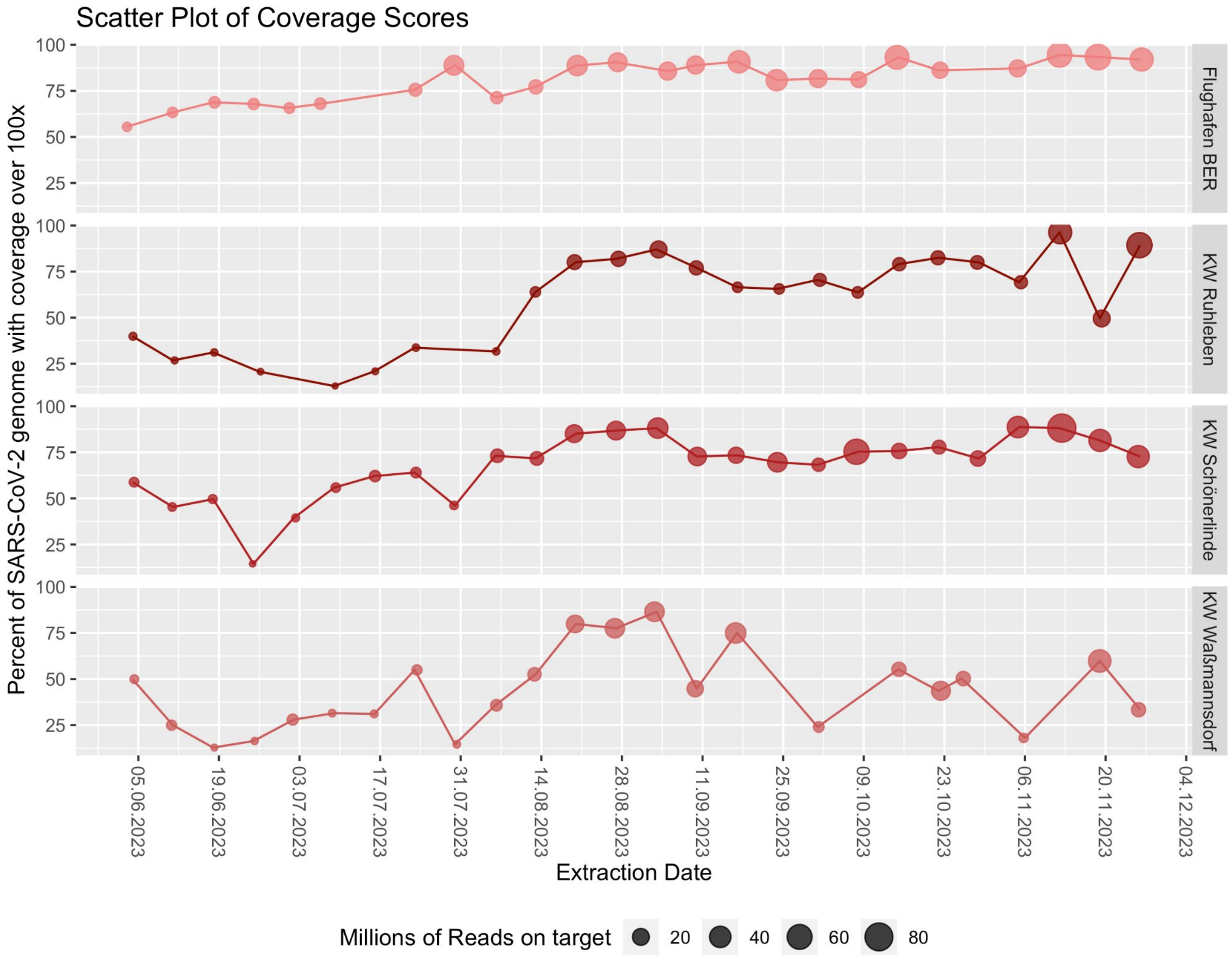

Figure S2. Scatterplot showing the depth of coverage per sequencing in each wastewater treatment plant. The coverage is calculated as the number of positions of the covid reference genome that are covered more than 100 times, the coverage score is is shown as the percentage. Notice much higher coverage score for the Berlin international airport during summer months.

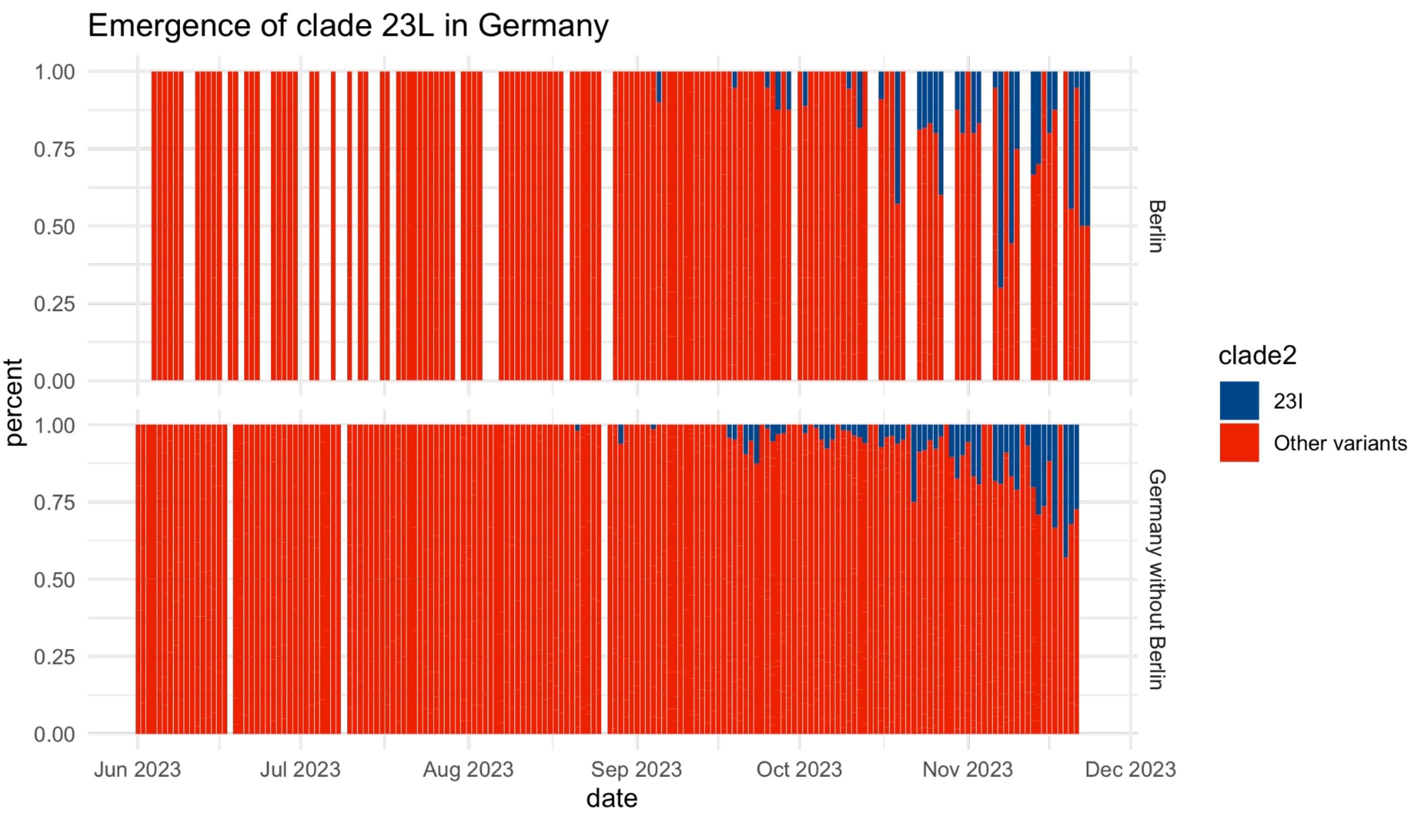

Figure S3. Barplot showing the emergence of 23L in clinical patien sequencing as deposited in GISAID.

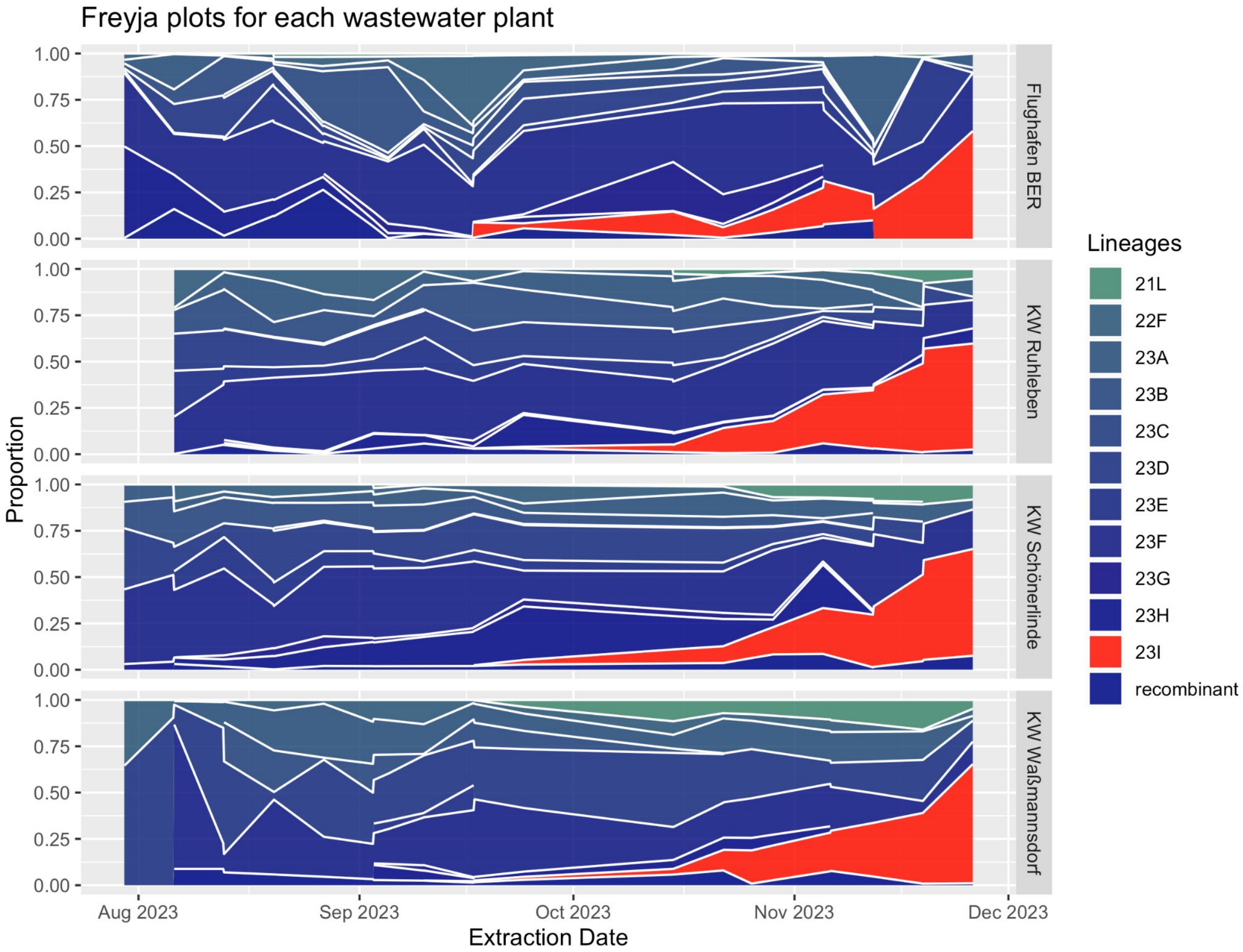

Figure S4. Freyja plots showing the emergence of linage 23L since August 2023 for each wastewater treatment plant. Notice the early detection of 23L in the Berlin International Airport.

Figure S5. Heatmap of fingerprint variants for each clade sequenced from the wastewater plant at the BER Berlin international airport.

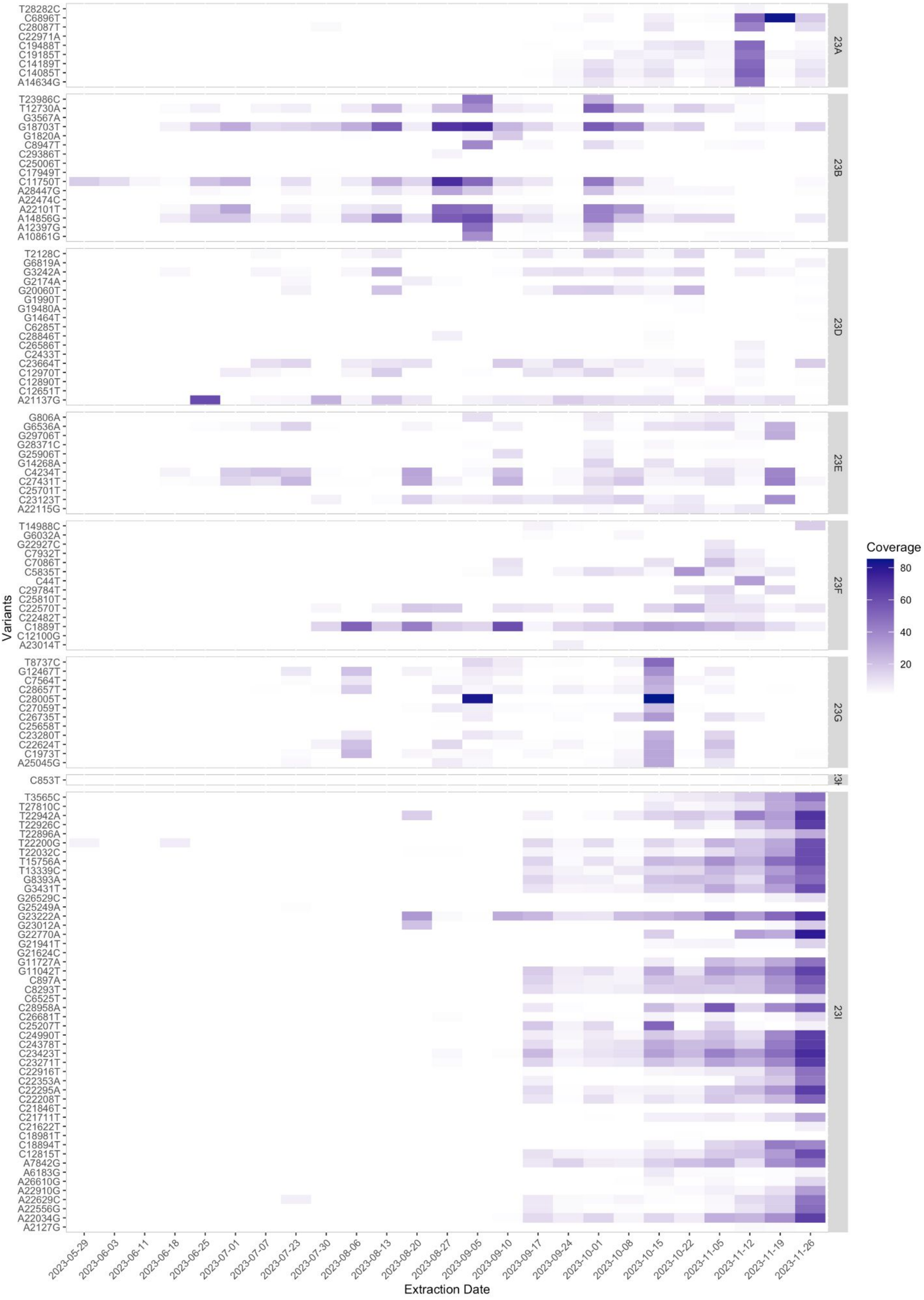

Figure S6. Heatmap of fingerprint variants for each clade sequenced from the wastewater plant of Ruhleben.

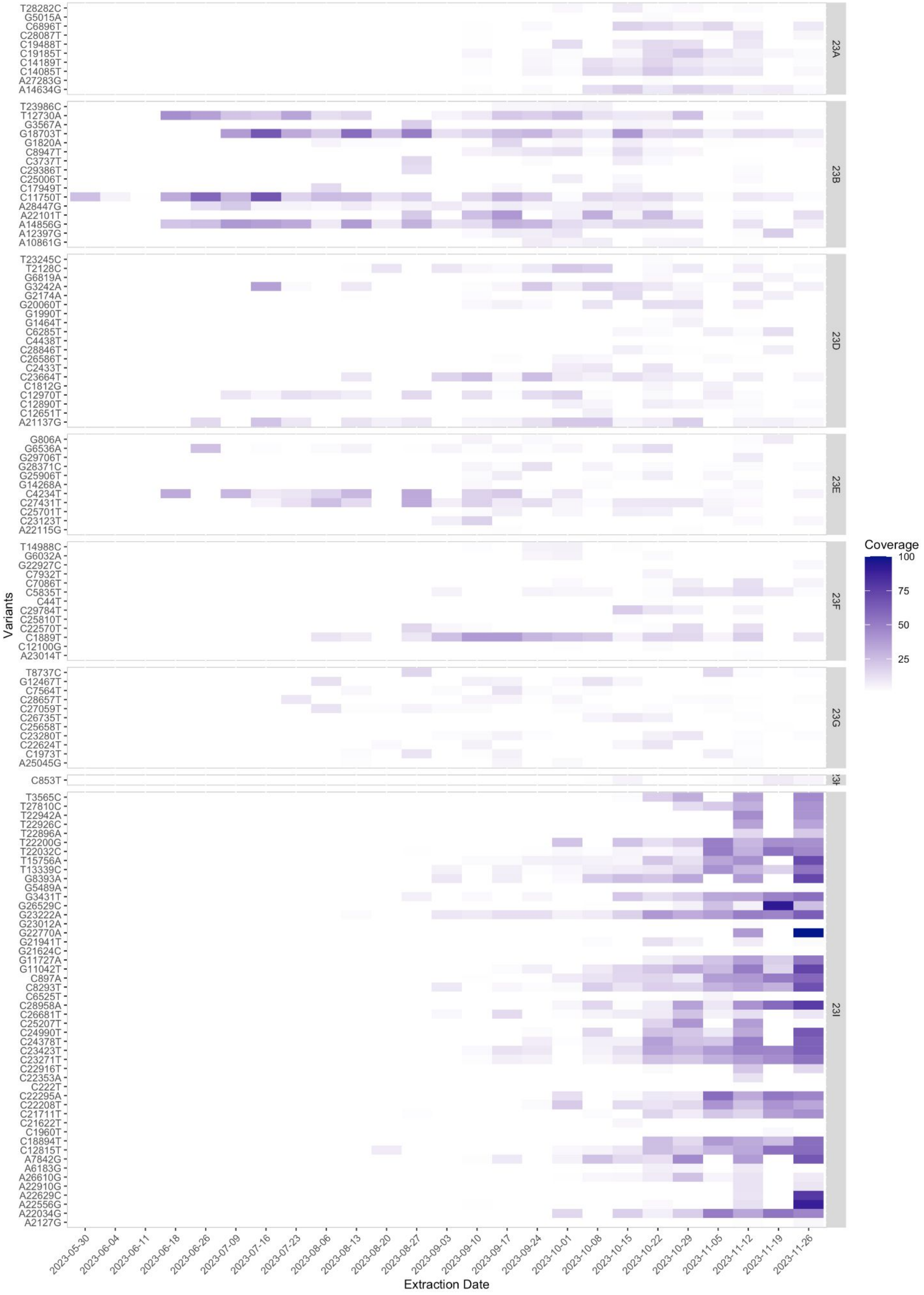

Figure S7. Heatmap of fingerprint variants for each clade sequenced from the wastewater plant of Schoenerlinde

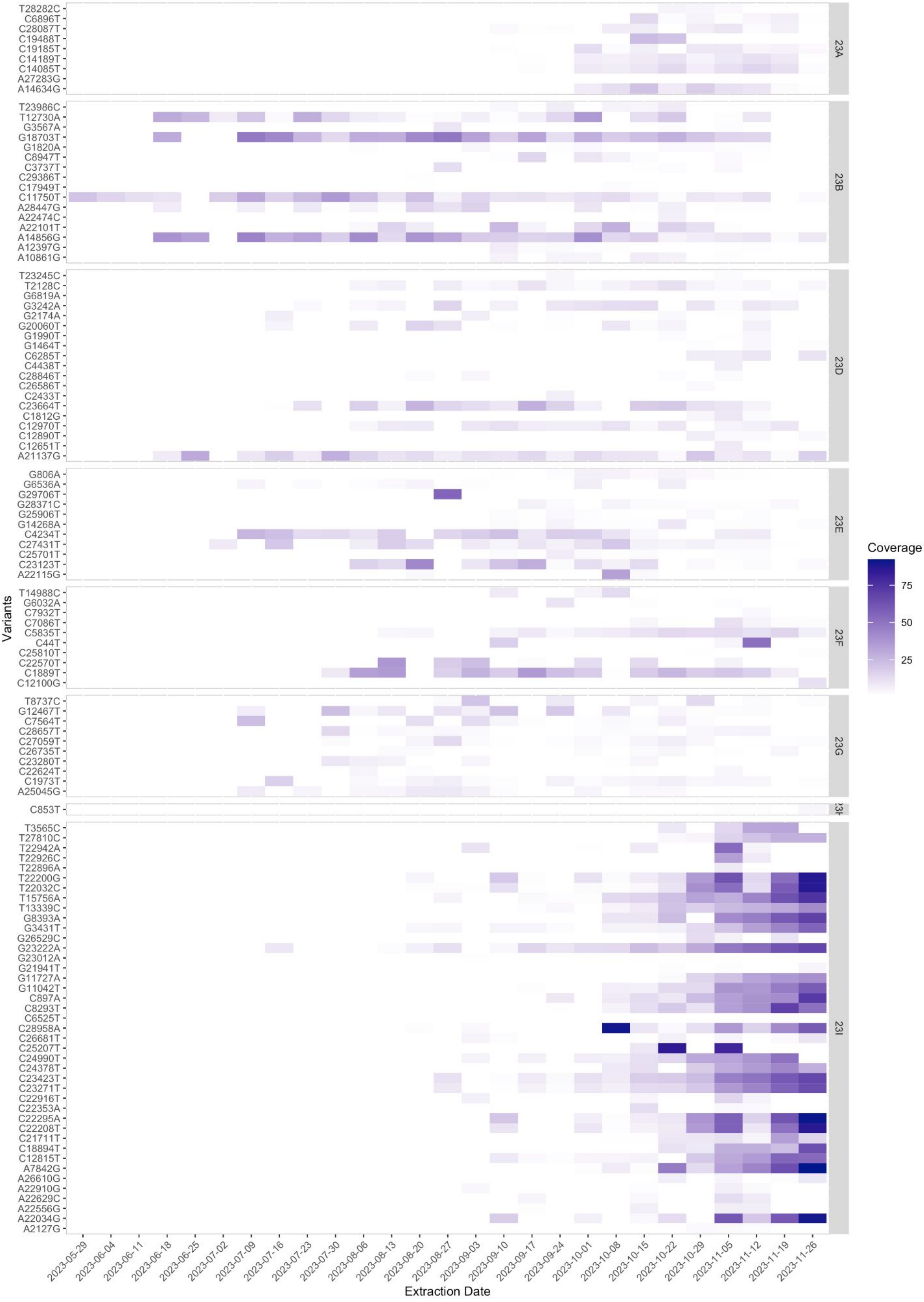

Figure S8. Heatmap of fingerprint variants for each clade sequenced from the wastewater plant Waßmansdorf.

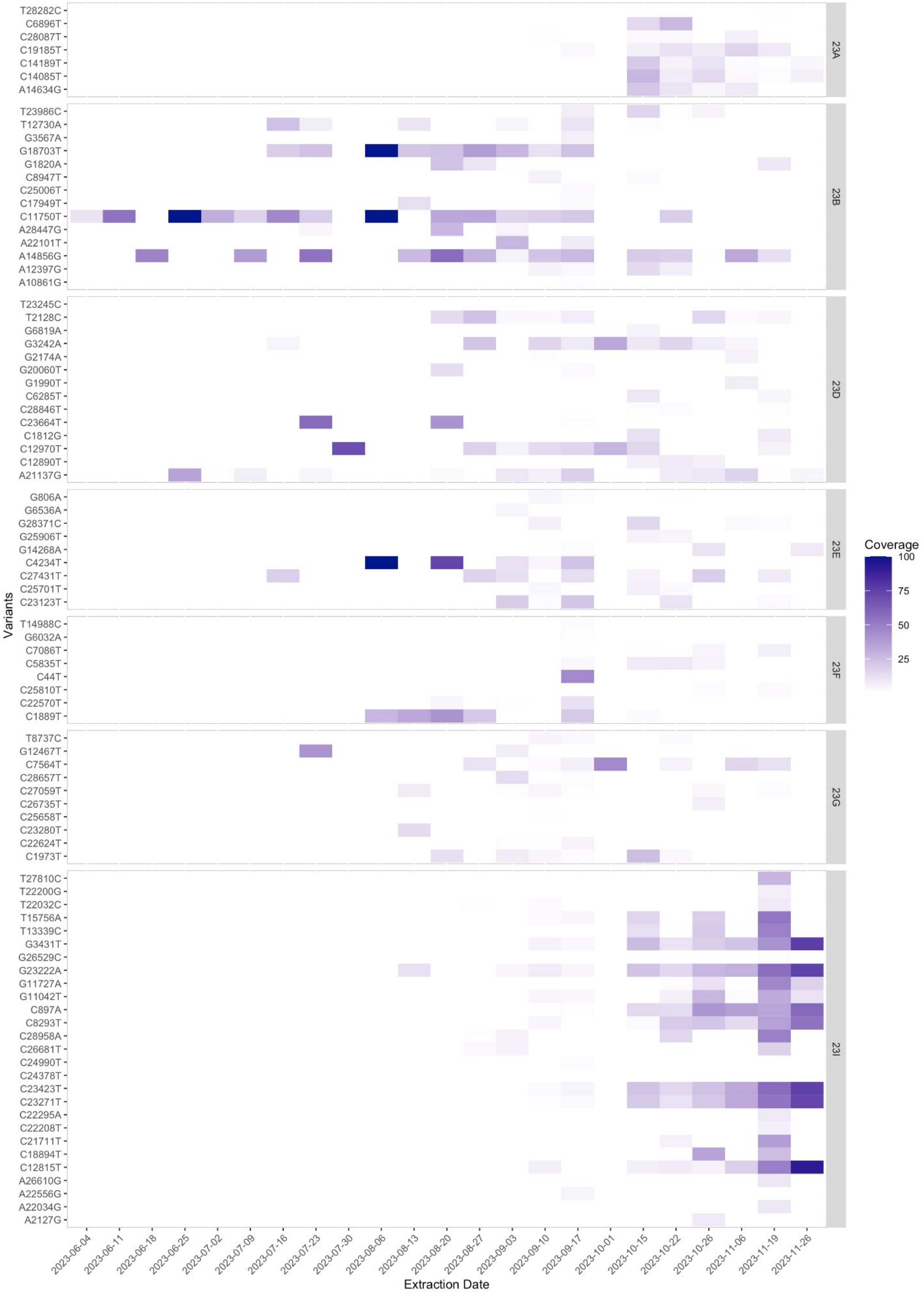

Supplement: Supplementary file 1 [file viruses-16-00102-s001.zip › viruses-2801332-supplementary.pdf]
